# A new variant of the electromagnetic field theory of consciousness: approaches to empirical confirmation

**DOI:** 10.3389/fneur.2024.1420676

**Published:** 2024-10-18

**Authors:** Wolfram Strupp

**Affiliations:** Department of Psychiatry and Psychotherapy, Asklepios Fachklinikum Brandenburg, Brandenburg an der Havel, Germany

**Keywords:** consciousness, electromagnetic field, mind–body-problem, qualia, information, binding-problem

## Abstract

There are various electromagnetic (EM) field theories of consciousness. They postulate an epineural EM field which, due to its binding properties, unifies the different neuronal information differences originating from various sensory and cognitive processes. Only through a real physical integration in space within this field could phenomenal consciousness arise. This would solve the binding problem mentioned in the philosophy of mind. On closer inspection, the electromagnetic interaction not only provides an explanation for the integrative property of the EM field, but also for the necessary differentiating contrasts of information. This article will take a closer look at the physical properties of a postulated EM field. It will also show how the problem of qualia in connection with emergentism could be solved by a new variant of EM field theory. If it can be clearly demonstrated that the postulated epineural EM field plays a decisive role in the origin of consciousness in addition to neuronal “wired” information processing, this also leaves less room for metaphysical assumptions that attempt to solve the binding problem. In experiments to prove the postulated epineural EM field by means of external electromagnetic manipulations, it can never be ruled out that these also have a direct effect on the “wired” neuronal signal processing. Therefore, on the way to proving the EM field theory of consciousness, an experimental method is needed that must ensure that external manipulations only affect the extensions of the EM field without directly influencing the neuronal network. A method will be discussed here that works with the shielding of EM fields instead of external electromagnetic stimuli.

## Introduction

### Philosophical background

Neuroscientific theories of consciousness that claim to solve the centuries-old mind–body problem, the ‘*hard problem*’, as the philosopher David Chalmers called it (1), are still not generally acknowledged. Is this because these theories are not yet sufficiently elaborated and developed? Or do physicalist theories that claim to explain consciousness and experience in neuroscientific terms have fundamental difficulties in gaining general acceptance? In the philosophy of mind, materialistic or reductive physicalist views (identity theories) are still challenged. The classical approach formulated by René Descartes (2), which posits an independent mental substance alongside the physical, such as an immaterial soul, is now rarely encountered. Nevertheless, so-called property dualism (3, 4) is frequently advocated, which basically assumes a single natural substance; however, the mental properties are still purported to elude known objective physical explanations. The argument of strong emergence (5) plays a decisive role here. Physicalist hypotheses on phenomenal consciousness (qualia, experiential qualities) are often countered with the argument that they cannot explain those properties, since experiential qualities are purely subjective in nature, whereas physics is objective.

A classic thought experiment in philosophy of mind is Mary’s Room by Frank Jackson (6): A brilliant scientist who possesses all available objective knowledge about the neurophysiology of color perception, but who has lived in a colorless room, is enriched by knowledgeable experience when she enters the colored environment. This is why the experience of colors cannot be explained by objective methods alone, and why physicalism is not sufficient. Against this, it can be argued that Jackson himself committed a category error in the Mary’s Room scenario, by requiring that a theoretical description or theoretical knowledge of a property must itself also participate in this property. But must it? Even in the case of an objective physical property, it is not necessary for the theoretical knowledge and corresponding formulae themselves to take on the physical property in question; theoretical knowledge about radioactivity does not have to be radioactive itself. And yet, its theoretical explanation is accepted. Nor would anyone think that a theoretical physical description of nuclear fusion must itself provide large amounts of heat. Therefore, theoretical knowledge and an objective explanation of consciousness and experience need not be synonymous with the experience itself nor substantially include the property of subjective experience. But in the case of mental processes, objective physical theories are analogously assumed to have precisely this deficit: Because an objective description remains precisely this, and cannot take on the subjective property itself, it is said to be unsuitable for capturing subjective processes (qualia). The Mary thought experiment and similar arguments often imply an inadmissible premise: Physics as a scientific discipline (research into natural phenomena and their description) naturally operates objectively. However, this does not mean that all physical processes *per se* must be exclusively objective in nature. In this case, the scientific discipline, which operates objectively, is equated from the outset, in whole or in part, with the properties to be investigated. For only if one already presupposes that consciousness differs entirely from the structure and function of physical states due to its subjective properties — something completely different in terms of quality — could one “prove” that an objective physical theory is abstracted from the subjective perspective. The conclusion is therefore already contained in the premise: a circular argument. Even if sufficient physical explanations were still lacking with regard to phenomenal consciousness, this does not allow the conclusion that these are not possible in principle, or even more so that it is inadmissible to approach this objectively. It seems as if some concepts of property dualism postulate that it is a kind of category mistake to look for objective explanations at all.

Just as the discipline of physics, which operates objectively, is wrongly equated *a priori* with physical natural phenomena (“everything that physics investigates has only an objective character”), the same occurs if erroneously presupposing the subjective to have exclusively subjective properties due to its phenomenal nature: Here, too, the equation/mixing of subjective operation (introspection) with the subjective property itself, which is supposedly not objectively accessible nor fully explicable, takes place *a priori*. However, if one therefore postulates that there are only exclusively subjective approaches to the phenomenon of experiential qualities, then the logical conclusion is that this does not apply to the possible qualities of other persons or beings, but only to one’s own experience (i.e., that of the postulating individual). For as soon as one makes assumptions concerning the qualities of experience in other beings, one already begins to operate objectively, i.e., to regard phenomenal consciousness objectively. However, since this cannot happen in the sense of a category mistake from the perspective of property dualism, one can only assume the existence of one’s own (!) qualia. Ultimately, however, this means solipsism. Other beings would always have to be regarded as philosophical zombies (3) or their presumed phenomenal experiences would only ever be an assumption. We could only ever accept on faith (without proof) that other people and beings (in relation to ourselves) also have experiences. However, such a solipsistic view would probably be out of the question.

Another main counterargument against physicalist views concerns the problem of “multiple realization” (7). It is claimed, for example, that the same mental property occurring in different beings, such as the sensation of pain, can obviously be realized in different neurobiological ways. Therefore, the mental property cannot be reduced to the physical. On closer inspection, this argument can be invalidated by considering the grain (8): Mental descriptions are simplistically presented as a coarse phenomenon such as “pain,” while finer differentiations are claimed for physical realizations. The supposedly same pain thus has many different physical realizations. If, on the other hand, finer differentiations are also applied to the mental descriptions (we know from experience that not all pain phenomena are the same), the assumption of multiple realizations can no longer be upheld.

Another central topic in the philosophy of mind and neuroscience is the binding problem (9). This concerns the question of how our brain combines different types of sensory (such as color, shape, movement, smell, etc.), cognitive and affective information processed in different brain regions to form a single, unified perception of an object or event; how these different sensory properties, which are spatially and functionally separated in our brain, are bound into a coherent perception. The very popular integrated information theory of consciousness (IIT) (10, 11) has been criticized by proponents of EM field theories of consciousness for lacking a truly integrating physical substrate [i.a. (12–14)] and thus not solving the binding problem. This crucial weakness is to be resolved through the electromagnetic field effect in a postulated integrating cerebral EM field (15, 16). However, further questions also arise here: What physical mechanisms are used for spatial integration within the electromagnetic field? Can the electromagnetic waves be considered as radiation? Can electromagnetic manipulations be carried out externally in experiments to demonstrate an integrating EM field without also having a direct effect on neuronal signal processing?

## Results

### The role of pyramidal cells in the formation of the EM field

Electromagnetic field theories of consciousness such as the conscious electromagnetic information (CEMI) field theory by McFadden (13, 17) or the general resonance theory of consciousness (GRT) by Hunt and Jones (12) share some statements with the IIT, but deviate from it in decisive points and thus open up new perspectives: According to IIT, experience and consciousness arise when there is a sufficient level of integrated information (value Phi, Φ). According to Tononi, there are special dynamic re-entry mechanisms in the thalamocortical system of the cerebrum that enable the necessary integration of differentiated information (18). This refers exclusively to neuro-synaptic pathways. Here, however, the aforementioned EM field theories differ in that they do not really regard these neuronal re-entry circuits, which only take place via discrete “wired” electrochemical signaling effects, as being physically integrated. Such a conventionally “wired” system, I would add, merely appears that way to an external observer. Therefore, a physically necessary integration must not be exclusively discrete-temporal via “wired” neural circuits, but must (additionally) be spatial via the postulated EM field. Since IIT has no real (i.e., physical) integrative component, Scott Aaronson (19) raises the criticism that a high phi value can also be achieved by abstract mathematical logic gates alone. Aaronson therefore criticizes IIT for potentially attributing a high degree of consciousness to systems that perform simple mathematical or logical operations, something that is at odds with our everyday intuition about consciousness. Aaronson’s example refers to special mathematical structures, such as low-density parity-check codes or simple logic gates. According to IIT, these systems could achieve high Phi values, a measure of integrated information and thus supposedly of consciousness. The problem here is that such systems are not considered conscious in the real world. They only perform basic mathematical or logical operations, and lack properties that we typically associate with consciousness, such as sentience or self-awareness. EM field theories of consciousness such as CEMI or GRT hold the view that real integration of information takes place spatially via a conjoint EM field. Conventional interconnections merely serve temporal, discrete signaling effects (which are of course also indirectly necessary for consciousness!). The distinction may be explained as follows: A neuronal network, the circuits of which could also be reconstructed with an old-fashioned mechanical calculator (no matter how unimaginably slow and of correspondingly large spatial extent), does not possess the property of real spatial integration, but only that of discrete temporal signaling effects. Therefore, such a network does not achieve complex consciousness (despite a mathematically high Phi value). Gottfried Wilhelm Leibniz’s (20) “windmill” thought experiment comes to mind here: If shrunk to the size of a microbe, one might enter a mechanical device (such as a windmill) and thereby observe its mechanical operation. However, even complete understanding of these mechanics would not reveal insights into consciousness or thought. The analogy argues that material processes cannot fully explain consciousness. Leibniz is to be agreed with here, because a network based exclusively on discrete processes of signal transmission lacks the real physical integrative component. Regarding the cerebrum, however, there is strong evidence of a spatially integrated overall electromagnetic field: An electroencephalogram (EEG) mainly records the dynamics of summed local field potentials of the cerebral cortex. The more postsynaptic potential fluctuations occur synchronously at the pyramidal cells, the higher the amplitude of the measurable sum potentials. These potential fluctuations and ion currents have electromagnetic influence and indirect induction effects, which have a wireless effect on more distant areas and can be measured outside the cranial cavity (by EEG and MEG). According to the most EM field theories, this dynamic EM field is identical to the phenomenal field of consciousness. McFadden (13) rightly states that there seems to be no obvious reason for synchrony in conventional neuronal processing (note: in relation to the synchronous oscillation of mainly local field potentials, which is measurable in the EEG and prevails to varying degrees), since neurons perform the same information processing regardless of synchrony. He refers to studies that prove the connection of mental phenomena with cortical synchronized oscillations rather than with membrane-bound spike actions [e.g., (21–23)] and sees this as an indication that mental phenomena are based on EM field effects. Synchronizations therefore only make sense in EM-based information processing. Later in this paper, I will discuss how these synchronizations (at the micro level) can be imagined with regard to information processing.

The pyramidal cells of the cortex seem to be ideal for a wireless electromagnetic remote effect. Their structure features a large cell soma and an extensive dendrite tree to which countless neurites (axons) dock synaptically. Large areas of the dendritic tree are depolarized by sodium ion influxes (excitatory postsynaptic potential), while the opposite field potential is formed extracellularly. The pyramidal cell thus functions as a dynamic “stepless” dipole (in contrast to axons, the all-or-nothing law does not apply here in the soma-dendrite area). The fluctuating potentials and associated ion currents are reminiscent of an LC-circuit: the pyramidal cells could therefore be both transmitters and receivers. It should be emphasized here that the electrical activity recorded in EEGs does not primarily represent the summed action potentials of axons (neurites), but rather the summations of the aforementioned local field potentials (LFP) between the cell soma and dendritic tree (both are non-myelinated!) of these specialized pyramidal cells (24). Pyramidal cells are the most numerous and morphologically largest type of neurons in the cortex. In addition to the presumed “antenna function” mentioned above, they are of course also conventionally connected: with correspondingly strong depolarizations, they also trigger axonal action potentials. Pyramidal cells have been shown to communicate conventionally (discretely), connected via known neuronal signal transmissions, but possibly also wirelessly via an electromagnetic remote effect.

### What kind of EM fields?

But what kind of electromagnetic fields are actually generated in the brain? We have known since the invention of the EEG and later the MEG that the cerebrum generates a dynamic of electrical potentials and magnetic fields that can be measured over several millimeters through the calvaria and epicranium. This allows us to hypothesize that these measurable potentials and currents may have an integrative remote effect with regard to consciousness. McFadden suggests an interplay of electromagnetic waves that are generated by the “firing of neurons.” We must examine exactly what is meant by this. If we refer to EEG and MEG measurements, it is true that these mainly arise from the summations of cortical local field potentials (LFP) and the associated ion currents, which are primarily related to the dynamics of excitatory and inhibitory postsynaptic potentials at the dendritic trees and somata of the cortical pyramidal cells, and less directly to axonal action potentials. If examining pyramidal cells and their extracellular ion gradients (local field potentials) and currents, one might make analogies with Hertzian dipoles, whose oscillating electrodynamics generate electromagnetic waves. But is this really the case? Do the cortical pyramidal cells or their extracellular potential differences and ion currents actually generate relevant Hertzian waves, i.e., electromagnetic radiation? Since the derivable potential fluctuations on the surface of the head predominantly appear in wave form, and specialized neuronal pacemaker circuits are also assumed to generate the different rhythms of the brain waves, analogies could be made with the carrier waves generated in radio traffic, which are modulated by the useful signals to be transmitted. According to McFadden (13), the interference of all these electromagnetic waves would produce an overall integration of the informative signals, a physical–spatial integration that can be “downloaded anywhere in this field.” Gamma oscillations (EEG terminology) in particular are mentioned when it comes to the integration of information from distant brain areas. Wolf Singer et al. have shown that synchronous oscillations occur, for example, in different parts of the visual cortex when a coherent image is perceived (25). The communication through coherence (CTC) theory by Fries (26), for example, explains the mechanism by which synchronous oscillations of different brain areas serve to connect information. A key element is phase coupling of oscillations occurring in different areas of the brain. It is assumed that these synchronously oscillating brain areas communicate with each other by allowing or blocking signals in a phase-dependent manner, and that information is transmitted selectively only between these synchronized areas. In these contexts, however, this theory assumes discrete neuronal (i.e., conduction-bound) signal transmissions, which are either facilitated or inhibited by the different oscillation frequencies and phase bonds. A long-distance electromagnetic effect is not assumed here. Although there is a spatiotemporal synchronization of different cortex areas, their integrative connection continues to occur via neuronal signaling pathways, not via spatial interactions of a postulated EM field. As mentioned, according to conventional views, such oscillations merely serve as a selection mechanism for the signaling connections between certain cortex areas. McFadden (13) claims that the spatial integration mechanism occurs via the interference patterns among individual electromagnetic waves within the overall electromagnetic field. This raises several questions: Can the oscillating LFPs that can be read in an EEG actually be described as electromagnetic waves in the sense of radiation? And if so, is the formation of interference from a large number of different individual electromagnetic waves really an integrative factor? After all, the respective information of the original input waves is no longer discernible in the resulting interference pattern. At best, the original waveforms can only be reconstructed if their information is still known. An interference pattern, which results from the interactions of several individual waves, does not involve the real integration of information, but merely corresponds to computerized processing. Despite occurring wirelessly, it would still only represent a further processing of information, as it could also take place via neuronal connections. This is because the new wave pattern simply combines two or more presumed electromagnetic waves (with individual frequencies and amplitudes) according to the classical interference model. This means that an EM field model based on the interference formation of electromagnetic waves does not fulfil the CEMI premise of genuine spatial integration of information. It is limited, albeit wirelessly, to informational wave processing in which information can even be lost. The result may contain less information than the input elements.

The General Resonance Theory of Consciousness (GRT) by Hunt and Schooler (12), also an EM field theory of consciousness, makes clearer reference to the local field potentials (LFPs) in the vicinity of the cortical pyramidal cells and sees the EM field arising from them as the seat of consciousness. The cortical oscillations resulting from summed LFPs are said to have an integrative and synchronizing effect on more distant cortex areas. Against traditional spike code theories of consciousness, which assume its physical substrate solely in the complexity of a network of membrane-bound signal transmission, the authors cite the following weighty considerations in favor of the EM field theory of consciousness in addition to the binding and integration argument: The paper by Hunt and Jones (27) cites several examples (including (28, 29)) of ephaptic couplings (electrical activations of cells that occur via electric fields rather than synaptic transmissions). This is followed by many examples that point to correlation and causality between different brain rhythms (neuronal oscillations) and mental states such as vigilance, cognition, attention, perception and short- and long-term memory [e.g., (30–32)]. These also include studies in which external electromagnetic stimulation was used [e.g., (33–35)]. According to Hunt and Jones, these compiled examples are intended to prove that cortical oscillations are not an epiphenomenon that merely occurs as an irrelevant by-product of synapto-neuronal wiring processes, but actually represent the causality between mental processes and the postulated EM field of consciousness. Exogenous electromagnetic manipulation of endogenous EM fields by means of transcranial magnetic stimulation (TMS), alternating current stimulation (TACS), direct current stimulation and deep brain stimulation therefore demonstrably have a direct influence on mental phenomena. According to the authors, they could not do this if the endogenous EM fields were only epiphenomenal - like the sound of a train whistle on a steam locomotive. In my opinion, however, this view would not entirely convince proponents of spike code theories: it seems unlikely that exogenous electromagnetic manipulations alone would have a targeted effect on the endogenous EM fields measurable in the EEG and MEG, while at the same time leaving out the membrane-bound ion gradients and voltage-dependent ion channels of the neuronal network. The “wired” neuronal signal transmissions therefore also inevitably change. Not only the “oscillome” would be manipulated, but also the functional connectome. Therefore, in my view, further methods (see below) are needed to prove the location of consciousness in the epineural EM field. Proponents of a spike code theory could also always argue that the cortical oscillations that can be visualized in EEG and MEG are merely a reflection of multiple synchronous neurosynaptic inputs to the pyramidal cells. After all, the LFPs, which are mainly derived from the summed ionic shifts of excitatory and inhibitory postsynaptic potentials, are a consequence of the inputs of axosynaptic spikes (action potentials). At this point it is also worth mentioning the EM field theory of Ward and Guevara (36), which assumes the seat of phenomenal consciousness in an electromagnetic field within a part of the thalamus. The cortex with its electromagnetically measurable oscillations, on the other hand, is seen more as a structure that carries out unconscious preprocessing. Although the authors advocate an EM field theory of consciousness, they surprisingly do not see the cortical oscillations that can be measured beyond the skull as the correlate of consciousness. This naturally raises the question of whether the thalamus, whose neurons do not exhibit such an extended “dipole morphology” as the cortical pyramidal cells, can produce such an effective epineural EM field. The GRT is described as panpsychistic: like the variant of the EM theory of consciousness presented here, it sees rudimentary consciousness already in the smallest charged elements of baryonic matter. The authors of GRT focus more on the concept of resonance and synchronization in general, without delving deeper into the specific physical mechanisms of electromagnetic interaction in the brain. However, the postulated electromagnetic resonance phenomena are not interpreted in terms of electromagnetic waves in the sense of radiation (Hertzian waves). A characteristic feature of GRT is the postulated intrinsic dynamics of the EM field in the sense of a self-organized resonance formation, which leads to coherence and synchrony of distant cortex areas via electromagnetic field effects. As with the CEMI theory, the postulated EM field of consciousness is thus not only ascribed a passive, experiential property, but it appears that the EM field also exerts active influences, although the authors do not explicitly name this as a correlate of free will. However, this raises the question of whether such postulated inductive resonance phenomena (field effects) can really be proven in relation to cortical oscillations. It could be argued that even the most strongly synchronized physiological oscillations (micro-level), namely the delta waves with their highest amplitudes, obviously require spike-code-mediated interconnection pathways for the transhemispheric synchrony and phase coupling (macro-level) observed in non-REM sleep (deep-sleep stage N4). This is because studies on split-brain patients have shown that in deep sleep, the delta waves in both hemispheres are no longer synchronized after commissurotomy (37). The situation is similar with generalized epileptic seizures, whose pathological, extremely synchronized oscillations (micro-level) no longer spread to the opposite hemisphere after a commissurotomy (38, 39). Even the strongest electromagnetic oscillations that the human cortex can generate do not show a sufficient active inductive effect for synchronization (at the macro level).

With regard to the physical character of the EM field of consciousness that I postulate, I suggest that the integrative electromagnetic field effect does not take place via waves in the sense of electromagnetic radiation, but via the basic phenomena of electromagnetic interaction: the Coulomb force, the electrostatic influence based on it, the magnetic influence with moving ions, and finally the induction force with moving magnetic fields. Electrical influence can be detected by recording electrical potentials in an EEG. Magnetic fields are generated by ion currents and can be detected by MEG. Since the magnetic fields do not remain static due to the dynamics of the ion currents, but the magnetic flux changes, induction forces could also arise from this, which in turn can cause changes in electrical potentials and ion currents. I do not rule out the possibility that this also leads to the generation of electromagnetic waves in the sense of radiation, but they cannot have a truly integrative function. The static Coulomb force already offers a core principle for information differentiation: two different charge polarities, positive and negative, which offer a fundamental differentiation, but this differentiation is naturally dependent on their integrative relationship to each other. With regard to EM theory, it can be said that it is the electromagnetic interaction that possesses precisely these physical properties that uniquely fulfil the concept of integrated information with regard to consciousness: The ions in the nervous system differ in their charge polarity (positive versus negative). This already offers a fundamental property of differentiation. However, the charges do not stand in isolation, but are related to each other through a long-distance spatial effect: The electric charge of an ion always acquires its differentiated specificity in relation to other charge carriers, whether in attraction or repulsion. The electrostatic forces and the electromagnetic influence occurring in dynamic processes up to induction thus best represent the integrating properties. There is therefore no need for a mysterious mental field of consciousness (40) with hitherto unknown properties, since the cerebral EM field perfectly fulfils the necessary requirements of differentiation and integration. This approach differs from McFadden’s EM field theory in that it does not situate the integration component in electromagnetic radiation (Hertzian waves), but in the electromagnetic interaction that charges exert statically and in motion, and not from the emission of real photons that arise from high-energy accelerated charge movements. In quantum field theory, a distinction is made between real and virtual photons. The Coulomb force through to the induction force are defined by the interactions of the virtual photons, so-called exchange particles (41). The integrative component of the EM field theory that I propose is therefore based on the interaction that is achieved via virtual photons. In this model, the informative differentiations are not abandoned. In a model that favors the interaction of Hertzian waves, interference leads to the phenomenon of superposition: the characteristics of individual input waves become incoherent when combined into a new resulting wave. As previously mentioned, this only involves the summation of information, not a real integration of differentiations. It may now be argued that coarsened and “smeared” potential differences are also obtained on the surface of the skull in the EEG, i.e., that there are also superimpositions in which the input signals merge into the resulting potentials. On the other hand, it can be argued that finer differentiation of the potential patterns is available with cortical recordings (high-density cortical EEG).

### An eventful electromagnetic field must be informative!

Why is it not possible to attribute a manifest phenomenal consciousness to an electrolyte solution that contains an abundance of charge carriers in ionic form, a plasma gas from a solar flare or a large, charged plate-capacitor from a physics lesson in the same way as a human cerebrum, even though there are plenty of electromagnetic charge carriers and field effects present? There may be countless electrical dipoles in an electrolyte solution, but these are so diffusely arranged and without complex relationships to each other that no complex informative contrasts occur. The charges largely balance each other out electrically. This means: many individual dipoles, each with only one bit of information (comparable to Tononi’s example of a digital camera containing many individual, independent photoelements). Such a system comprises only independent individual elements, with very little integrated information. In addition, the individual electrostatic and electrodynamic differentiations are simultaneously neutralized by opposing processes. Accordingly, there is no complex phenomenal consciousness. Even a charged plate-capacitor, regardless of the number of homogeneously separated charge carriers, would only have a single information difference, and thus, despite a possibly strong electrostatic field effect, only the smallest unit of integrated information (only that of a single difference), but still the smallest effective unit. This simple potential difference would have the information content of one bit. At this point, however, it should be mentioned that the spatial integration of even the most complex charge differences alone does not signify consciousness if no changing dynamics take place. Pockett therefore used the term *spatiotemporal* patterns (16). What does the cerebral cortex offer in contrast to an electrolyte solution and a plate-capacitor? The cortex therefore contains this “forest” with the “antennae” of the non-myelinated sections of the cell soma and dendrites of the pyramidal cells, whose electrical potential fluctuations and ion currents exert field effects on the environment. If these cells lose their ion gradients in the event of severe damage and an isoelectric zero line appears in the EEG, one can assume unconsciousness (in this case, irreversible) comparable to an electrolyte solution. In a generalized tonic–clonic (grand mal) seizure, on the other hand, dynamic hyper synchronizations of oscillating postsynaptic potentials of many pyramidal cells and the corresponding field potentials occur. During such a seizure, the EEG shows generalized spike–wave activity. Here, the hypersynchronized potential oscillations can be compared with a charging and discharging plate capacitor in terms of integrative information content. This means the strongest electrical contrast, but also—due to the absence of complex differentiation — the coarsest of all information states. There are also no complex differentiations here. There is also unconsciousness here. Such coarsened electrical polarities occur during epileptic seizures, but also in a softer waveform during the deep sleep phase (non-REM sleep). The information content decreases, and with it conscious experience. To summarize: both electroneutral dispersion as well as excessive electrical homogeneous contrasting of ions result in a loss of information—and thus a loss of consciousness. Non-REM deep sleep/coma and generalized seizures can therefore not be experienced; only the pre- and post-phases are experienced. So what characterizes the electrical states of the brain during experience and dreaming (REM sleep)? Put simply, the electrical charge carriers must form a dynamic of diverse but (spatially) integrated complex patterns that lie between the extremes of homogeneous contrast and diffuse dispersion. This can also be observed in the EEG. Beta and gamma waves in particular, which are present during the conscious waking state (and in dreams during REM sleep), show summation potentials of dynamic multiple synchronizations. In other words, there are no hypersynchronizations in which neuron clusters oscillate extensively in unison as in generalized epileptic seizures, but also no blurring of the ion gradient dynamics to uncertainty, as when each individual pyramidal cell oscillates on its own without reference to others, or even in brain death states when ions without gradients would move diffusely isoelectrically.

### Qualia: how are they realized in electromagnetism?

In contrast to IIT, the postulated EM theory offers the proposed solution of the spatial remote effect by means of an electromagnetic field with regard to the actual integration of information into a physical system. But how can we imagine the presence of all qualia in the electromagnetic field? If we now consider both the information effect through dynamic electrical potential differences and the integration effect through field effects in accordance with EM field theories, the following seemingly bold statement can be made: All possible qualia are potentially contained in the fundamental force of electromagnetic interaction. The capacity for consciousness or experience is therefore another basic property of electromagnetism. This statement seems almost magical. But why should not what applies to the objective states of matter also apply to the subjective world? After all, it is the properties of electromagnetic interactions that determine the diversity of matter and thus of the objective world (42). If only electrically neutral bodies of mass existed, there would be no diversity of matter, because this is decisively determined by electromagnetic interaction. Overall, electronegativity contributes significantly to the diversity of chemical compounds and materials that we observe in nature and in synthetic processes. It is a key factor in understanding chemistry and the diversity of matter. This means that electronegativity potentially contains all the diversity of the objective world. No-one seriously claims that, in the sense of strong emergence, the multitudinous diversity of the phenomena of matter is somehow inexplicable. There is a consensus that this property can be reduced to basic physical forces, in particular “electronegativity.” According to the EM field theory proposed here, the same holds in the subjective world (Table 1): the dynamic, complex, manifold combinations of charge carriers and their field effects make up phenomenal consciousness.

**Table 1:** Electronegativity and the diversity of the world.

Objective world: Mass Charge (and its forces) - > Chemical diversity of matter	Subjective world Mass Charge (and its forces) - > Diversity of qualia

For phenomenal consciousness, we therefore do not need any additional natural properties alongside the already known basic physical forces, since its properties and modes of action are already contained in them, particularly in electromagnetic interaction. But how can we explain how the necessary information, the differentiations, are available in connection with electromagnetic integration?

### The electrolyte solution as a pool of all possible qualia

**Figure 1:**
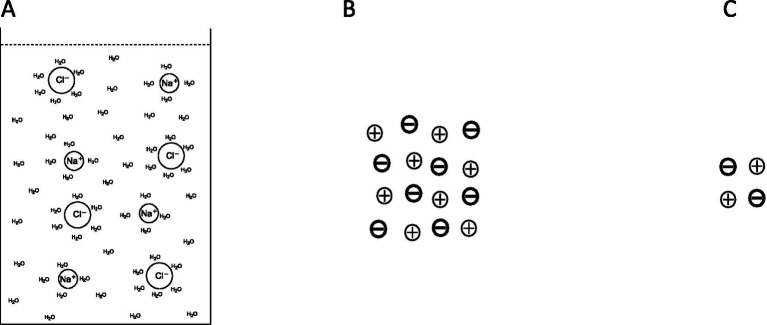
In **(A)**, a saline solution contains hydrated sodium cations and chloride anions. There are no outwardly effective ion gradients, nor are there any net ion currents, as these are neutralized electromagnetically by opposing dynamics. Panel **(B)** shows positive and negative charges arranged in such a way that no net gradients arise. From an informational point of view, there are no effective differentiations here. These could also be replaced by the arrangement of ions in **(C)** without changing the information content.

**Figure 2:**
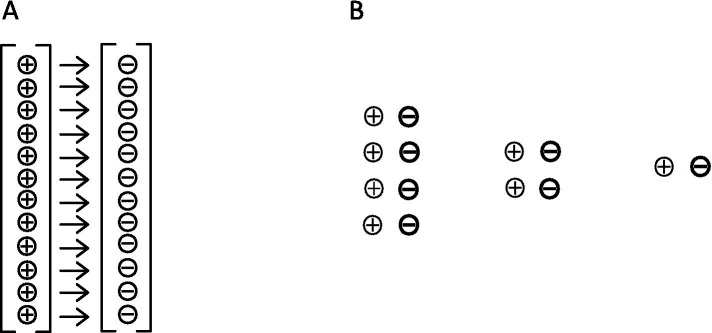
Panel **(A)** shows a plate capacitor. This separates charges and generates a strong homogeneous electric field. Although countless charge carriers are involved, in terms of information there is only a single differentiation. **(B)** Shows other arrangements of charges in which complete separation of the polarities results in a simple informative difference.

**Figure 3:**
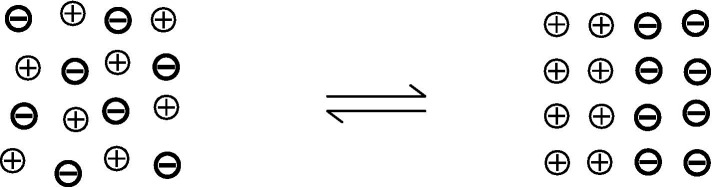
The process of changing from uncertainty to the greatest possible charge separation. Both extreme states: uncertainty and uniform polarity.

**Figure 4:**
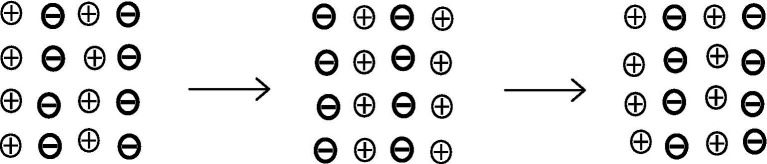
A dynamic of the homogeneous alternation of polarities is shown here, as occurs, for example, in generalized epileptic seizures. For the sake of simplicity, only snapshots of apical charges of the local field potentials (LFP) are shown.

**Figure 5:**
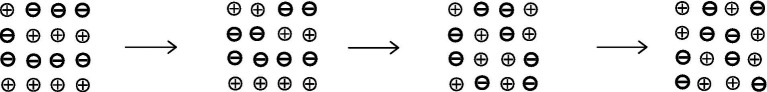
Here the schematic represents snapshots of apical charges of local field potentials (LFP), which have greater differentiation than the aforementioned uncertainty of an (electrolyte solution) and that of a coarse, uniform-polarity separation (in hypersynchronizations).

Let us imagine an electrolyte solution (Figure 1) in which all ions are evenly distributed and hydrated. Here, the informative differentiation elements, the electrical charge carriers – cations and anions – and the integrating electromagnetic field effects are already present (electrostatics and magnetostatics up to induction). Nevertheless, consciousness cannot be assumed here, as it has been verified that a brain shows no consciousness after the collapse of all ion gradients, comparable to an electrolyte solution. But we could say that all possible qualia are potentially present in this outwardly isoelectric electrolyte solution, because potentially (!) all possible informative contrast patterns are present, which the ions in a vital brain can also have. However, it could be understood that they are all superimposed in the electrolyte solution, i.e., electrostatic and electrodynamic differentiations are simultaneously neutralized by opposing processes, so that ultimately only a dispersion of individual hydrated ions is found: uncertainty, from an informational point of view. In this case, unconsciousness could be understood as the result of ubiquitously “overlapping” electromagnetic differentiation patterns, which are equivalent with informational uncertainty. An initial differentiation with information gain is possible as soon as a first simple charge separation occurs — similar to a plate capacitor (Figure 2). For an electrolyte solution in the biological field, these are charge separations on membranes: Qualia uncertainty has hence become an initial bipolar differentiation. Since all processes of conscious experience are dynamic, the process of changing from diffuseness to the greatest possible charge separation is shown in Figure 3. However, the information gain is only one informative difference. Neither extreme state — i.e., uncertainty or uniform polarity—is compatible with consciousness. In generalized epileptic seizures, but also in delta deep sleep, dynamic hypersynchronizations occur among the oscillations of local field potentials (LFP). The informative differentiation here corresponds to that of an alternating simple homogeneous charge separation (Figure 4). Figure 5 illustrates how the qualia could be gradually “modelled out” via more complex dynamic differentiation. These examples illustrate what the constellations of further differentiations and complexifications could look like.

### The problem of emergentism

In contrast to weak emergence, in which the emergent properties can in principle be explained by the interactions of the system components, strong emergence is characterized by the assumption that the resulting properties or behaviors are in a certain sense new or unexpected and cannot be fully explained by an analysis of the individual system components (5, 43). The question remains unanswered as to what exactly is responsible for the emergence of phenomenal consciousness as the complexity of the interaction of the physical system components increases. In his criticism of strong emergence, Jaegwon Kim (44) implicitly alleged hidden dualism. His concerns related to the fact that if emergent properties — as postulated in theories of strong emergence — are not reducible to the properties of their constituent parts and have *their own causal efficacy*, this could lead to a kind of dualism.

The problem of (strong) emergence in phenomenal consciousness can be resolved if the potentialities of all possible qualia in the electromagnetic interactions are already regarded as a given. In the objective world, the situation is no different with regard to the occurrence of possible chemical properties of matter: As mentioned above, we know that electromagnetic interaction is responsible for the complex chemical properties and diversity of matter. It is not problematic to reduce this to the physical properties of electromagnetism. Just as the “shaping” of the objective world depends decisively on electromagnetic interaction, so does the “shaping” of the subjective world.

### The problem of specialized qualia

If a certain neuronal state A in the brain is associated with the qualitative impression of the color red, and another neuronal state B with the color blue, the following question arises: What exactly should be so special about these states that that they represent the experience of “red” and “blue” respectively?

The problem can be solved in the context of the EM field theory of consciousness as follows: It is not the case that a neuronal state A (or B) in isolation represents the experience of the color red (or blue). If we assume that all possible qualities are present in the electromagnetic interaction: Then a neuronal state A is associated with the experience of red if the neuronal states for all other colors and — even more so — for all other qualities of experience are also realized as a contrast in the background. The cerebral neuronal states that are necessary for a certain quality of experience, e.g., the perception of a color, are therefore not realized separately and not in isolation, but always in connection with the realization of the other qualities. It is precisely this relativity that forms the qualia. Parallels can also be drawn here with Tononi’s parable of the photodiode and its perception of light and dark (10, 11); accordingly, conscious experience depends on the fact that during the currently experienced phenomenon, such as the change from a white to a black screen perceived by a human brain, there must also be a connection to a multitude of other possible ways of distinguishing. In contrast to a photodiode, a human brain has a multitude of other means of differentiation, such as the ability to differentiate between yellow, green, or red screens, as well as many other qualities. This is what makes the distinction between light and dark contentful and conscious. It follows that, with regard to consciousness, spatiotemporal integration in the cerebrum is not limited to one particularly activated area alone, but that the non-dominant “repertoire” of all other possible qualities must also always be related. Even if the brain’s visual system is especially active during concentrated visual conscious perception, the other sensory and associative areas must always be integrated as a repertoire and contrast reference. If we could manage to keep the visual system active in the cerebrum, but at the same moment deactivate one or other sensory system for a short time, this should also have an effect on the quality of the visual experience. It is not just a matter of deactivating the respective sensory input and the primary cortical sensory center, but also of inhibiting the associated higher association area in particular. As a result of the impoverishment of the repertoire of other perceptual qualities, the visual experience would be significantly less contentful and less phenomenally conscious. It therefore requires the integration of all other possibilities as repertoire and contrast in the background. To emphasize this once again: Of course, there are highly specialized areas of the cortex that are responsible for the most diverse qualities of experience. These areas are also highly specialized for the respective sensory modalities. Nevertheless, the isolated activation of the neuronal representation for a single color, for a single quality of experience, is not sufficient. It requires the entire cerebral EM field with all potentially contained qualities, which provides the contrast to the activated, emphasized quality.

I would also like to address the argument of inverted qualia: Jackson (45) wondered whether two people who objectively view one and the same color hue might not have subjectively different color impressions. Theoretically, one person could have the impression of green, and other of red. If we now apply this question to the EM field theory, in particular to the proposed connection that all possible qualia are potentially contained in the EM field, then we can conclude that the respective person experiences the color impression depending on how highly differentiated the neuronal structures and networks are, how highly differentiated the EM field is. If one brain works with less differentiation, there is also less color differentiation available than in another brain that has more differentiation possibilities. The subjective impressions therefore differ in this respect.

### A clinical-experimental detection method

This version of an EM field theory of consciousness presented here postulates the necessity of a spatial–physical integration of information, which of course also includes the significance of the temporally discrete signal interconnections in the integration of information. The “final stretch,” however, is the spatial and physical integration of the information differences, as this is the only way to establish comprehensive relationships between the different information contrasts. This is where electric charge carriers come in, as they already have these integrating properties due to their spatial influence and induction effects. Many electromagnetic manipulation methods have already been applied to the human brain, both non-invasive (e.g., transcranial electrical and magnetic stimulation) and invasive (cortical and subcortical electrodes). If the aim is to change a presumed epineural EM field through artificial external influence in order to bring about corresponding changes in experience and behavior, it cannot be ruled out with certainty that this will not happen without simultaneous manipulation of the intraneuronal electromagnetic conditions. In other words, it is never possible to manipulate only the presumed epineural EM field, i.e., to act only on the extracellular local field potentials, without also manipulating the cerebral network circuitry, the “wiring.” Even if, according to Pockett (46), the cell soma and the dendrites of the pyramidal cells have fewer voltage-dependent ion channels than the axon and the axon hillock, it cannot be ruled out that an external change in extracellular ion gradients will also result in a change in the ion currents in the chemically sensitive ion channels of the chemical synapses, which will affect the excitability of the pyramidal cells — and thus also the transmission of signals in the neural network. Consequently, another way must be found, which can successfully demonstrate the effectiveness and manipulability of an epineural electromagnetic field without having a primary and direct effect on the nerve cells and their network. In this context, the exciting question now arises: What is the spatial distribution and range of the field? Is there an integrative electromagnetic spatial long-distance connection encompassing the two cerebral hemispheres? We know that the cortices of the two cerebral hemispheres do not merge histologically. Both hemispheres are connected to each other by nerve fiber bundles (axons) of white matter, so-called commissures. The main commissure is the corpus callosum. In patients with therapy-resistant epilepsy, these commissures are even today in some cases surgically separated in order to inhibit the transhemispheric transmission of hypersynchronous axonal firing rates. In complete split-brain patients, all conventional interhemispheric connections are therefore severed. According to Tononi’s IIT, there should therefore be two separate units of consciousness. It should be noted that in investigations of split-brain patients, it was initially assumed that after commissurotomy there were two largely independent units of consciousness. But then the question arose: How is it possible, for example, that split-brain patients can speak intelligibly, even though speech is primarily controlled by the left hemisphere? Usually the left hemisphere should control the motor cortex in both hemispheres in order to coordinate the muscles involved in speech production. However, studies have now shown that these speech centers are also active together in the right hemisphere. But how does the right side learn from the left, and how are they kept synchronized when the corpus callosum is severed? Pinto et al. (47, 48) discussed the possibility that interhemispheric synchronization continues to occur via subcortical pathways and enhanced ipsilateral pathways, among others. The corpus callosum consists of a bundle of approximately 200 million myelinated axons. McFadden (13) suspects that not only neuron-guided signals are transmitted via the corpus collosum, but that electromagnetic waves, which are supposed to be generated by the synchronized firing of neurons, are also transferred from one hemisphere to the other. However, the white myelinated substance is not considered to be directly involved in the generation of the summed cerebral LFPs measurable in the EEG (this occurs in the grey matter), so it seems questionable whether the corpus callosum is directly involved in the transmission of wireless electric field effects between the hemispheres. Rather, only wired neuronal signal transmissions should take place within the commissures in the sense of conventional connections.

The fact that split-brain patients show deficits in terms of contralateral information transmission, yet overall give the clinical impression of a single conscious agent, could speak in favor of the hypothesis of spatial electromagnetic integration. Even if hypersynchronous epileptic activities are no longer transmitted axonally to the contralateral side following a full callosotomy, this does not fundamentally argue against a remaining integrating electromagnetic remote effect, so that there remains an experienced unity of consciousness — rather than two distinct fields of consciousness within a single cranium. If the EM field theory is correct, it should be possible to achieve a separation of consciousness, or at least a restriction, simply by electromagnetically decoupling both brain hemispheres without severing nerve tissue. If spatial electromagnetic integration (field effect) is a prerequisite for consciousness, this would have to encompass both hemispheres. Electromagnetic field isolation would therefore have to be provided for a certain period of time, for example by inserting an electrically shielding membrane into the interhemispheric gap. This would interrupt or at least significantly impede the presumed long-distance electromagnetic effect between the two hemispheres. With such experimental electromagnetic shielding, impairments, perhaps even a fission of consciousness and experience could be expected — or possibly even disordered states such as dissociation or delirium. In a theory of consciousness that exclusively propagates a conventional neuronal circuitry, however, such “Faraday isolation” would have no influence; in a postulated electromagnetic field theory, however, it would have a decisive influence. It is worth mentioning the pioneering consideration by McIver (49), who refers to earlier failed shielding experiments with, e.g., gold leaf shields on the surface of the cortex: A superficial cortical shielding that merely interrupts electromagnetic field effects in the direction of the cranial calotte is unlikely to be sufficient. It must be ensured that a transhemispheric or interlobar interruption of the postulated endogenous EM field is really guaranteed, as this exerts its integrating effect three-dimensionally toward the center. The problem of realizability must be taken into account here. Such an experiment could only take place for a short time as an adjunct to an ongoing neurosurgical procedure during which the test subject remains awake. If the insertion of such a membrane did not result in any change in experience and behavior, this would challenge the assumption of an integrating overall electromagnetic field. However, if such an electromagnetic split-brain were to cause a significant disturbance, perhaps even more pronounced than in patients with a tissue callosotomy, this would strongly support the EM field theory with its assumption that the wealth of information must be spatially integrated by electromagnetic remote effects and that conventional circuits alone are not sufficient. Such a shielding membrane could be inserted not only within the interhemispheric fissure, but also in the lateral sulci, for example. As already mentioned, brain tissue should not be damaged in the process, i.e., no neuronal network connections should be severed; only the electromagnetic coherence of the presumed cerebral epineural EM field should be interrupted or at least impaired. If an integrating overall electromagnetic field is discerned, this would not only provide impressive verification of the EM field theory of consciousness, but also a strong indication that no additional supernatural power, spirit or soul is required additional to the physical prerequisites. This would be a decisive step toward solving the mind–body problem.

## Discussion and conclusion

EM field theories of consciousness are a promising approach to solving the mind–body problem. The binding properties of the EM field unify the various neuronal information differences originating from different sensory and cognitive processes that are distant from each other in the cortex. McFadden metaphorically postulated a “mind–energy-dualism,” in which energy refers to the “immaterial” extension of the EM field (13). This should explain a certain autonomy for free will. In the variant of EM field theory postulated here, the epineural field is not located in an “electromagnetic wave cloud”: Due to the fact that the binding component of the field is not assumed to occur in an interaction of electromagnetic waves in the meaning of radiation (Hertzian waves), but predominantly in the area of “virtual photons,” which play the decisive role in the Coulomb force through to electromagnetic induction, the electrical charge carriers remain more “materially” involved as polar core components of the informative differentiations. The EM field is not “free” in a cloud but arises from the narrower electrostatic and electrodynamic effects within the folded “electrolyte carpet” of the cerebral cortex. This EM field theory of consciousness emphasizes precisely these peculiarities, which can be derived from the cerebral wetware (a term coined by the novelist Rudi (50)): A three-dimensional field of hydrated ions whose electromagnetic effects arise from the dynamics of a complex gradient formation on membranes. I therefore call this variant within the EM field theories of consciousness the Electromagnetic Ion Field Theory of Consciousness (EIFT), as it takes particular account of the ions, whose gradients, as already mentioned, act as informative differentiation components: “the difference that makes a difference.” In this context, I would like to return to the role of cortical oscillations: As previously described, General Resonance Theory (GRT) addresses these cerebral waves (“resonances”) and ascribes both differentiating and integrating functions to them. Hunt (51) sees the integrative component in a “slowest shared resonance frequency (SSR),” which serves as the basis for the synchronization and integration of neuronal activities. This SSR (theta and alpha waves) should act as a kind of base frequency whose synchronization extends the furthest in space and in which faster oscillations (such as beta and gamma waves) are embedded. This idea is based on the principle of hierarchical oscillations, in which slow waves synchronize larger networks, while faster waves support more local, specialized processes. In contrast to McFadden’s CEMI theory, no interference principle of electromagnetic waves is used here; the faster cerebral waves would not be eliminated. However, this raises further questions: In relaxed subjects with eye closure without higher cognitive tasks, in most cases there is an alpha-wave dominant EEG. Here, faster waves can certainly be found in parallel in the same cortex areas with high-resolution or cortical recordings. However, theta waves, which are synchronized over extended cortex areas, only occur with reduced vigilance or pathological slowing. Otherwise, they are only found in localized form in healthy people with normal vigilance. In highly vigilant, healthy subjects with eye opening, the alpha waves are usually suppressed and there is a beta wave dominance. It can hardly be assumed that slow waves continue to exist in the background. This is because alpha and theta waves have higher amplitudes and represent a stronger synchronization of neuronal oscillations at the micro level, i.e., more neurons are recruited for simultaneous oscillation than with the fast waves. So if they are present to a relevant extent, they are also prominent and not invisible in the background. According to GRT, however, this could mean that in the absence of the postulated alpha and theta waves as integrating, spatially extended basic resonances, only localized resonances are present. However, this would mean a fragmentation of consciousness, which is atypical for a vigilant, attentive state of consciousness that processes sensory stimuli. The EIFT presented here does not see the mechanism for the integration of consciousness in synchronized oscillations: As already mentioned, it does recognize the importance of macro-level synchronizations (i.e., between different, even distant cortex areas) in optimizing neuronal information processing, as outlined by Fries (26) in his CTC theory, but this is neuronal “wired” information processing. Fries also attributes the alpha and theta waves more of an inhibitory or masking effect. From an electromagnetic field point of view, this is not contradictory: alpha waves mean coarsening within the cerebral EM field compared to the faster waves, but this can also mean greater contrast (at the micro level) with regard to informational differentiation. The electromagnetic integration effect in EIFT does not take place via synchronized resonances (see GRT) or waves in the Hertzian meaning (see CEMI), but via the basic EM field forces (Coulomb force, influence, induction). This means that charge movements with different frequencies also interact electromagnetically with each other. The EIFT also assumes that the entire EM field-generating cortex is necessary for the formation of consciousness, whereby there are dominant areas, but these must always be in relation to the rest of the repertoire. This is also in line with IIT, which considers this remaining repertoire (see analogy of the photodiode) to be essential. When considering IIT, however, there appear to be contradictions: On the one hand, the photodiode analogy underlines that in addition to the mechanisms of the current perceptual quality (screen with the alternating colors light and dark), the mechanisms that stand for all alternative perceptual and memory qualities must also be integrated. On the other hand, however, when describing the “dynamic core,” the dynamic network which, according to IIT, stands for the current conscious perception and which does not include the entire thalamocortical system, the entire repertoire no longer appears to be present in the integration field. This may be due to the fact that, according to IIT, it is sufficient that only processed information from the repertoire reaches the “dynamic core,” because the entire IIT system is based on processed information flows due to the discrete signal transmission mechanisms. It therefore seems only logical that for a truly physical integration of information in space, as demanded by EM field theories, GRT advocates a hierarchical oscillation principle in which faster local oscillations are embedded in slower waves in a nested manner. Here, the subunits are not eliminated as a result of information processing, but remain undamaged. EIFT, in contrast, does not propose a circumscribed, central dominant field of consciousness within the entire cortex area, but considers the entire cerebral EM field as a field of consciousness. The parts that are particularly dominant in consciousness could be the areas with the faster oscillations, while the areas with the slower waves form the repertoire contrast. However, this does not refer to delta waves or extended (!) theta waves, as these have a diminishing effect on consciousness due to their hypersynchronization (micro-level) as a result of excessive electromagnetic coarsening (doziness to deep sleep). However, all cortex areas remain electromagnetically related to each other, there is no decoupling. The areas with the faster waves do not necessarily have to be at the same frequency and phase coupling in order to be integrated. However, as already mentioned, such synchronization (macro-level) is useful for information processing between these areas.

*EIFT* can be described as reductive-physicalistic, since, firstly, no substance other than the physical is required (no soul or immaterial spirit), and secondly, no strong emergence is required that leads to a further property of phenomenal consciousness that can no longer be reduced to the physical. Even if the subjective side of consciousness does not appear to be objectively accessible in the experiential meaning (!) due to its purely private access, this does not mean that it remains objectively and physically inexplicable, nor that it cannot be reproduced. As Hales and Ericson (52) have also pointed out, electromagnetism is the decisive fundamental physical force. This also means that one could, in principle, construct an artificial conscious wetware. In this sense, the form of EM field theory postulated here could also be regarded as a type of gradual panpsychism (53). It should be emphasized here that this does not refer to a view that favors an independent intellectual mind, before the origin of matter, which subsequently took on a material form. In gradual and evolutionary panpsychism, the mind arises “from below” without a pre-existing intellectual template or an intended goal. The laws of evolutionary theory apply. The differentiating components required for consciousness, which make complexity possible in the first place, namely the polar charge carriers, are only provided by matter. An immaterial, subjectively perceiving spirit, if it existed, would also require the prerequisites for the realization of information and integration. As far as our world is concerned, however, this is best provided by electromagnetic interaction. In contrast to GRT, which represents a gradual panpsychism on all levels of matter due to the postulated universal resonance principle, EIFT is limited to the parts of matter from which electromagnetic charges and their effects emerge (in the case of antimatter, there could well also be “positromagnetism”). In the case of a form of matter consisting only of neutrons, for example, there would be no minimal form of rudimentary consciousness. The EIFT thus represents a gradual electromagnetic panpsychism/pan-experientialism.

Susan Pockett sees her EM field theory as a vehicle theory according to Atkinson’s categorization, while she classifies McFadden’s CEMI theory and Tononi’s theory of integrated information as process theories (14, 54). But should not a sufficient theory of consciousness encompass both components? The electromagnetic interaction, whose forces emanate from charged particles, is the vehicle of the property of consciousness. But without informative processes within this vehicle, consciousness with the qualia spectrum remains undifferentiated and merely on a potential level. Especially since the electromagnetic interaction with its informative basic elements of consciousness, namely the charge polarities, provides the ideal conditions for both vehicle and process. Clinical experience shows that extreme electromagnetic processes such as a generalized epileptic seizure, in which the electromagnetic fields occur at the highest synchronized strength, are not compatible with consciousness. The deep sleep states associated with hypersynchronizations (delta waves) also indicate that a loss of informative complexity is associated with a loss of consciousness. Even generalized diffuse alpha activity (alpha coma), which can occur after severe brain damage, is associated with unconsciousness (55, 56). Therefore, in addition to the electromagnetic vehicle component, the informational process component is also required, which makes the dynamic complexity of the EM field possible in the first place.

The question of whether there is something akin to volitional autonomy within the overall ionic EM field, which is derived from the integrated overall effect, cannot be answered from this. However, the possibility need not be ruled out. It is quite possible that the cerebral EM field has a feedback effect on the neuronal circuit mechanisms. However, it should first be confirmed that the cerebral EM field actually represents the seat of consciousness.

## Data Availability

The original contributions presented in the study are included in the article/supplementary material, further inquiries can be directed to the corresponding author.
